# Assessing the utility and efficacy of e-OSCE among undergraduate medical students during the COVID-19 pandemic

**DOI:** 10.1186/s12909-022-03218-9

**Published:** 2022-03-08

**Authors:** Sarra Shorbagi, Nabil Sulaiman, Ahmad Hasswan, Mujtaba Kaouas, Mona M. Al-Dijani, Rania Adil El-hussein, Mada Talal Daghistani, Shumoos Nugud, Salman Yousuf Guraya

**Affiliations:** 1 Department of Family and Community Medicine and Behavioural Science, College of Medicine, University of Sharjah, Sharjah, United Arab Emirates; 2 Baker/ IDI Heart and Diabetes Institute, 75 Commercial Road, Melbourne, VIC 3004 Australia; 3 Clinical Sciences Department, College of Medicine, University of Sharjah, Sharjah, United Arab Emirates

**Keywords:** Clinical assessment, e-OSCE, Psychomotor skills, COVID-19, Medical students

## Abstract

**Background:**

The outbreak of coronavirus disease 2019 (COVID-19) and its quick progression to a global pandemic has urged medical schools to shift from didactic to distance learning and assessment approaches. The quality of clinical training and assessment have been jeopardized due to the regulatory restrictions and potential hazards to human lives. The aim of this paper is to evaluate the utility and efficacy of an electronic Objective Structured Clinical Examination (e-OSCE), which attempted to transform the format of a face-to-face OSCE to an e-OSCE.

**Methods:**

We conducted three end of clerkship e-OSCEs for final year medical students in Surgery, Medicine and Family Medicine using the teleconferencing application of Microsoft Teams (MST). The e-OSCE blueprint included the assessment of all clinical skills except physical examination and procedural skills. Examiners supervised e-OSCE from the college campus, while all students were remotely assessed through the MST channels. During the exam, the students stayed in their specified MST channel and examiners rotated across all students. The utility and efficacy of e-OSCE was evaluated using a self-administered questionnaire for students, examiners and e-OSCE team.

**Results:**

The data analysis showed that 93.4% students and 84.3% examiners agreed with the quality and process of e-OSCE. Similarly, 83.6% students and 98% examiners agreed with the seamless organization of e-OSCE. As many as 45.9% students and 74.5% examiners agreed that e-OSCE was close to real life practice. Approximately one fifth of students and one third of examiners preferred e-OSCE over the face-to-face OSCE. The analysis of qualitative data generated the themes of e-OSCE structure and technology. While majority of participants were satisfied with e-OSCE, students were concerned about examiners’ training and e-OSCE contents. Examiners and e-OSCE team recognized the paper-less, tech-savy, fast and reliable format of e-OSCE.

**Conclusion:**

During and beyond COVID- 19 era, e-OSCE is a strong substitute to standard OSCE for assessing clinical competence except for physical examination and procedural skills. The planning and implementation of e-OSCE reflects an ingenuity in the assessment of clinical competencies of medical students.

**Supplementary Information:**

The online version contains supplementary material available at 10.1186/s12909-022-03218-9.

## Background

The Corona-Virus Disease 2019 (COVID-19) pandemic has substantially disrupted the optics of several aspects of communities worldwide, including healthcare systems, academics and medical education [1]. The emergence of COVID-19 and its rapid progression to a global pandemic has urged many countries to employ emergency lockdown plans and to harness social distancing policies. Following the directive by the Accreditation Commission for Graduate Medical Education (ACGME), many schools and universities around the world have adjourned their educational missions, with an adverse effect on an estimated 1.5 billion learners worldwide [2].

In the higher education, the sudden and paradigm shift from on-campus to distance learning approaches have posed a challenge to faculty, students, university academic management and Information Technology (IT) experts. Reciprocally, front-line physicians have rapidly adapted to the evolving transformations in the delivery of health care. From the academic perspectives, medical educators have shown tremendous resilience towards the development of an absolutely virtual educational climate [3]. Virtual education pertains to a teaching environment where instructors and students are dissociated by time or space, or both [4]. In virtual education, instructors deliver course contents through a range of learning management applications, multimedia resources, videoconferencing, breakout groups for students’ engagements and peer assisted learning [5]. Likewise, during the COVID-19 pandemic, assessment of cognitive and meta-cognitive domains has been adapted by technology-enhanced learning using several commercially available applications such as Blackboard, Zoom, MS Teams (MST) and Google Hangouts Meet [6]. These applications employ blogging, curation, collaboration, communication, infographics, mind mapping, podcasting and videoconferencing. All such features tend to promote interactivity among students that invariably leads to life-long and experiential learning.

Restricted by the constraints of the COVID-19 pandemic, medical schools have attempted to transform all educational activities to online learning formats. This requires a dedicated teamwork that would continuously monitor and evaluate the transformational process. Educators should also antedate the challenges and plans on how to overcome the ongoing challenges and hurdles. Some of these challenges include the overstretching of the learning management systems, internet interruptions, and limited skills of faculty members or instructors in using online teaching technologies [7]. Moreover, training in the clinical environment and assessment of psychomotor skills have emerged as eminent challenges for medical educators and policy makers worldwide [8]. During the clinical years of medical school, the format of assessment needs to be scrupulously planned to maintain its validity, reliability, and feasibility. The conventional assessment methods in medical colleges including written exams, clinical and practical skills must be re-envisioned.

The objective structured clinical examination (OSCE) is a well-recognized tool for the assessment of a plethora of clinical skills and competencies including medical professionalism, behaviours, attitudes, data analytics, history taking and problem solving domains [9, 10]. During the COVID- 19 pandemic, the face-to-face format of the OSCE would not be possible because of its associated high risk of disease transmission among students, examiners, and organizers and even patients. Henceforth, we have witnessed a transfer of the traditional face-to-face OSCE to an e-OSCE to ensure social distancing and to safeguard the health of students, faculty, and patients [11]. This e-OSCE has been forecasted as an innovative assessment tool since it utilizes an entirely online application for summative assessment of clinical skills, except for the assessment of psychomotor and procedural skills [12]. Unfortunately, during the pandemic, conducting and maintaining the quality of OSCEs to determining the competency of medical schools’ graduates is an unprecedented challenge [11]. This challenge falls into an uncharted territory for medical schools and this situation is further complexed by a lack of structured guidance and regulations by local health authorities and medical educators.

In order to curtail personal contact for reducing the risk of exposure of the COVID-19, all medical schools adjourned clinical clerkships of undergraduate and postgraduate programs [3]. On 15^th^ March 2020, due to the suspension of clinical clerkships and electives by the Ministry of Education of United Arab Emirates (UAE), the College of Medicine (CoM) University of Sharjah (UoS) aborted the clinical training of the fourth- and fifth-year undergraduate medical students. To avoid the potential delay of the graduation of the final year students who had passed their previous clinical clerkships, the assessment committee and the CoM council adjudicated to conduct the end of clinical clerkship assessment through the available online platforms. Subsequently, we conducted e-OSCE for the final year medical students at the end of their last clinical clerkships. To our knowledge, so far no e-OSCE has been reported from the Middle East and North Africa (MENA) region. The specific research questions of this paper were aimed to; 1) evaluate the utility and efficacy of e-OSCE during the COVID-19 pandemic at the CoM UoS; 2) compare the students’ assessment performance between the traditional face-to-face OSCE and e-OSCE. The analysed data will serve as a valuable source for other academic institutions that are endeavouring to establish similar clinical assessment program in high stake examinations.

## Materials and methods

### Study settings

In May 2020, we planned, implemented, and evaluated the utility and efficacy of e-OSCE at the CoM UoS. The MBBS program at the COM UoS is a six-year integrative and problem-based learning program that spans over three phases: phase I (foundation year), phase II (preclinical years one, two, and three) and phase III (clinical years four and five). In year four, students rotate though four 10-week clinical clerkships in surgery, medicine, paediatrics and obstetrics and gynaecology, while year five students attend three 10-week clinical clerkships in surgery, medicine, and family medicine. Each end of clerkship assessment includes a written and a clinical examination including the Direct Observation Clinical Encounter Examination (DOCEE) using real patients and OSCE using standardized patients. Completing the clerkship clinical training and all the clerkship assessments is a prerequisite for sitting for the final MBBS examination.

### Planning and implementation of e-OSCE

The CoM made an e-OSCE team of a director, nine clinical tutors, and two information technology personnel. Three e-OSCEs were organized at the end of clinical clerkships of Surgery, Medicine and Family Medicine for final year medical students via the teleconferencing application of MST. The e-OSCEs were run across three consecutive days using MST software due to its simplicity, quality audio and video interfaces, breakout room features, and low-cost. Furthermore, MST provides opportunities for synchronous online monitoring and recording of students’ activities during and between stations by direct observations. A great majority of the e-OSCE examiners were hospital physicians who had previously supervised face-to-face OSCEs. The e-OSCE team conducted a series of online training sessions for all examiners. In addition, the team also organized a mock e-OSCE for 105 students over two days where members of the OSCE team acted as examiners. Feedback from the students and examiners helped the team to identify areas for improvement; mainly related to noisy background since the examiner were placed in the same room. This issue was solved by procuring high quality microphones and headphones of Logitech™ video capture connectivity technology with 720p resolution. The Logitech™ C270 delivers smooth long-distant video calls for clear communication and allows to record videos in HD quality. Additionally, Logitech™ fluid crystal technology ensures sharper pictures, richer colours, and clearer sound in real-world conditions.

### Contents of e-OSCE stations

The contents of e-OSCE stations were based on a blueprint that was designed to assess students’ communication, history taking and clinical reasoning skills and decision-making (problem-solving) ability to manage common medical problems (Additional file 1: Appendix 1). The checklists of the selected e-OSCE stations, previously used in the CoM, were transferred to google forms for their usage by examiners on iPads. We were not able to evaluate physical examination and procedure skill via the e-OSCE format. In history taking stations, examiners role played the given clinical scenarios. Concurrently, the examiners would challenge the students about possible diagnosis and management plans. In some stations, examiners would ask students to interpret X-rays, electrocardiogram, videos, or images that were tagged with clinical scenarios.

### Design of e-OSCE

**Fig. 1:**
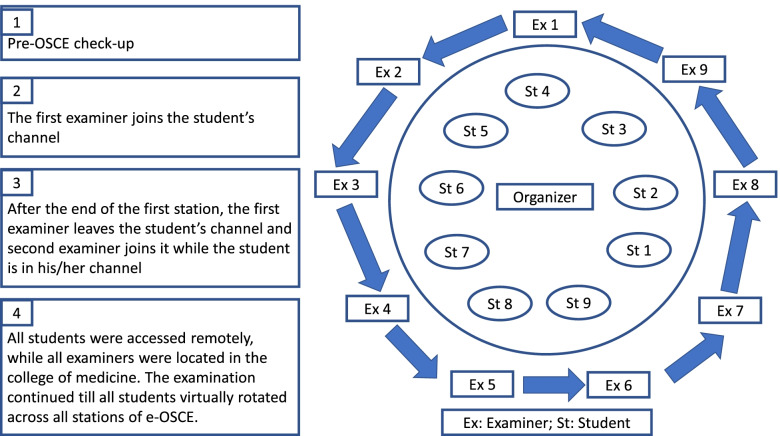
An illustration of the key steps in the organization of e-OSCE. All students were accessed remotely, while all examiners were located in the college of medicine. The examination continued till all students virtually rotated across all stations of e-OSCE

**Fig. 2:**
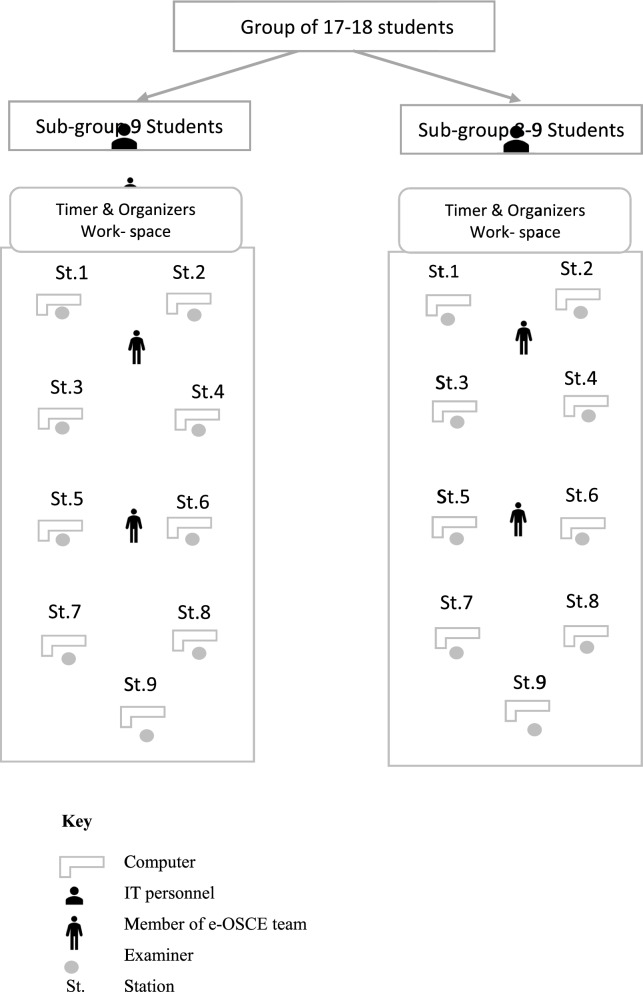
A layout of panels for examiners, and organizers during e-OSCE

The e-OSCE blueprint included skills of history taking, clinical reasoning, communication and, decision making. Assessment of physical examination and procedural skills were not included. At the CoM, the examiners were distributed into two parallel groups, in two separate large halls by maintaining social distancing. Each group had nine examiners who were supported by three organizers and one information technology staff. All students were remotely assessed through MST channels. Student were asked to have their cameras and microphones continually on to promote academic integrity. While the students stayed in their specified channels, examiners rotated across all students (Fig. 1). The panels of examiners were physically present in the CoM, while students were assessed remotely from their own locations. Each examiner's panel was supported by the organizers of e-OSCE team (Fig. 2).

### Organization of e-OSCE

Each of the three clinical clerkships of surgery, medicine and family medicine had 35–36 students. The students were divided into two groups of 17–18 students each. Each group was further divided into two sub-groups (A and B) and (C and D). The A and B sub-groups were assessed at the same time by two parallel panels of examiners on the same set of nine stations. The time allocated for each station was seven minutes plus one minute between stations. The cycle of e-OSCE for first two sub-groups (A and B) was completed in 72 min. Following that, the second sub-groups (C and D) were assessed by the same panels of examiners using new sets of stations with different contents. Availability of examiners and organizers in the campus was essential to facilitate coordination among panels and to hasten the resolution of arising technical issues. A total of 54 examiners participated in the three end-of-clerkships e-OSCEs.

### Microsoft teams and channels

The e-OSCE team created two MST panels including nine examiners in each panel. Within each MST one unique channel was created for each student. Each panel assessed nine students in each round. Each channel was labelled by the student’s name and UoS identity number.

Before the start of the e-OSCE, the organizer engaged students by MST to their respective channels, started video recording and confirmed their identification. Then students were directed to show a 360° environmental scan by their own cameras. Unlike the traditional OSCEs where students rotate around the stations, in e-OSCE, the students remained in their respective channels while examiners navigated from one channel to another according to a pre-planned students’ circuit. Once the examiners were connected with the students through channels, the organizer would set an alarm to indicate concurrent start of all stations. Each examiner then read instructions for the students in 30 s, followed by the specific task. The examiner would ask the students to ‘leave the channel’. The process was repeated nine times in the first round. There was a 15-min break before the start of the second round.

### IT support

IT support was crucial to ensure efficient and smooth implementation of the e-OSCE. The IT personnel provided continuous advice and training to organizers and examiners on the MST. We used 22 desktop computers, each supported by webcam and headsets with two laptops as a backup. All computers were connected to the UoS land line Internet connections.

### Grading of e-OSCE stations

The performance of students was recorded synchronously through robust checklists which were developed on google forms in iPads. The checklists had pre-determined rating scales and each examiner was instructed to submit his evaluation checklist online at the end of each station. At the end of e-OSCE, the google forms are computed, collated, and analysed electronically and then the final review was undertaken by the director and members of the e-OSCE team.

### Reducing the risk of infection

Since examiners and organizing team were present in the CoM, all necessary precautions recommended by the Ministry of Health were considered to mitigate the risk of disease transmission. These measures included minimizing the number of people in one room, keeping a safety distance of at least 1.5 m between examiners in all directions, wearing masks to cover nose and mouth, and sanitizers. Furthermore, gloves, caps, aprons, and face shield were available for examiners and organizers, if needed.

### Security of e-OSCE

Two measures were taken to reduce communication between the first and second group of students. The first measure was to call the second group of students for an online briefing one hour before the first group has completed their OSCE evaluation. After the briefing, the second group of students were called into their respective MST channels by organizers. Students would remain in their assigned channels till the end of their examination. The second measure to ensure the security of e-OSCE was the introduction of a new set of stations with equivalent contents and face validity that were used for the second group of students.

### Assessment of e-OSCE

We assessed e-OSCE by administering online self-administered questionnaires using google forms to students, examiners and e-OSCE team (Additional file 1: Appendices 2, 3, and 4). The questionnaires were developed by the members of e-OSCE team, were revised and improved by a group of experts in medical education. This process of instrument development was completed by piloting the questionnaires on respective cohorts. The respondents essentially pointed out the need to enrich questionnaires with more issues for logistics, Internet bandwidth and technical support for e-OSCE. These observations were incorporated into the final versions of questionnaires.

During the pre-COVID era, a comprehensive face-to-face OSCE was a standard practice which included stations for the assessment of a full range of clinical skills and competencies. Simulated and standardized patients, video-based assessment, and clinical skills using manikins and plastic models were used. All manned stations were graded by examiners on paper-based checklists.

### Statistical analysis

The data of the survey questions were extracted from the online survey software (e-survey pro) to the MS Office Excel sheet and was analysed using the Statistical Package for the Social Sciences (SPSS) by IBM™ version 27, USA. The quantitative analysis primarily consisted of descriptive statistics (frequency and percentages) and the responses to the open-ended questions were reviewed and common themes were identified by researchers. For the comparison of academic performance of students between the 2019 (pre-COVID, face-to-face OSCE) and 2020 (during COVID, e-OSCE), we calculated mean and median of students’ grades using the t-test if the data were normally distributed and Mann–Whitney U test if the data were skewed. We followed Saldana approach for qualitative data analysis [13]. Using a descriptive approach, we coded datum primary contents which was then categorized into groups. This process generated broader understanding which led to the generation of different themes. The data was further verified and cross-checked by all authors.

Research bias was mitigated by frequent discussions and by reaching a consensus by referring to the original data.

## Results

### Quantitative data

A total of 105 students from surgery II, medicine II and family medicine clinical clerkships appeared in e-OSCE. Out of 105 invited students and 54 invited examiners, 61 (58%) students and 51 (94.4%) examiners responded to questionnaires as shown in Tables 1 and 2. Most students (93.4%) and examiners (84.3%) perceived that the level of tested knowledge was fair, while 45.9% students and 74.5% examiners strongly agreed/agreed that e-OSCE was close to the real-life practice. Likewise, 83.6% students and 98% examiners strongly agreed/agreed that e-OSCE had seamless organization. Approximately, one fifth of the students and one third of examiners preferred e-OSCE over the traditional face-to-face OSCE.

**Table 1:** Analysis of students’ responses about the organization, quality and evaluation of e-OSCE

Questionnaire statements	Surgery (*N* = 15)	Medicine (*N* = 21)	Family Medicine (*N* = 25)	Total (*N* = 61)
**1. Overall, the tested level of knowledge and skills**				
Too difficult/difficult	0	1	1	2 (3.3%)
Fair	15	19	23	57(93.4%)
Too easy/easy	0	1	1	2 (3.3%)
**2. e-OSCE was closer to real life practice**				
Strongly disagree/disagree	1	3	4	8 (13.1%)
Neutral	8	4	13	25 (41%)
Strongly agree/agree	6	14	8	28 (45.9%)
**3. e-OSCE was well organized**				
Strongly disagree/disagree	0	2	0	2(3.3%)
Neutral	5	3	6	14(23.0%)
Strongly agree/agree	10	16	19	45(73.8%)
**4. e-OSCE was conducted smoothly**				
Strongly disagree/disagree	1	1	0	2(3.3%)
Neutral	2	2	4	8(13.1%)
Strongly agree/agree	12	18	21	51(83.6%)
**5. Preferring e-OSCE over the traditional face to face OSCE**				
Strongly disagree/disagree	9	9	13	31(50.8%)
Neutral	5	7	5	17(27.9%)
Strongly agree/agree	1	5	7	13(21.3%)

**Table 2:** Analysis of the examiners’ responses about the organization, quality, and evaluation of e-OSCE

Questionnaire statements	Surgery (*N* = 17)	Medicine (*N* = 16)	Family Medicine (*N* = 18)	Total (%) (*N* = 51)
**1. Overall, the tested level of knowledge and skills**				
Too difficult/difficult	0	1	0	1 (2)
Fair	14	13	16	43 (84.3)
Too easy/easy	3	2	2	7 (13.7)
2. **e-OSCE was closer to real life practice**				
Strongly disagree/disagree	1	1	1	3 (5.9)
Neutral	4	4	2	10 (19.6)
Strongly agree/agree	12	11	15	38 (74.5)
**3. e-OSCE was well-organized**				
Strongly disagree/Disagree	0	0	0	0
Neutral	0	1	0	1 (2)
Strongly agree/agree	17	15	18	50 (98)
4. **e-OSCE was conducted smoothly**				
Strongly disagree/disagree	0	0	0	0
Neutral	0	1	0	1 (2)
Strongly agree/agree	17	15	18	50 (98)
5. **I am interested to be called as an examiner in future for similar OSCEs**				
Strongly disagree/disagree	0	0	0	0
Neutral	1	2	0	3 (5.9)
Strongly agree/agree	16	14	18	48 (94.1)
6. **Preferring e-OSCE over the traditional face to face OSCE**				
Strongly disagree/disagree	7	4	5	16 (31.4)
Neutral	8	7	0	15 (29.4)
Strongly agree/agree	2	5	13	20 (39.2)

### Qualitative data

**Fig. 3:**
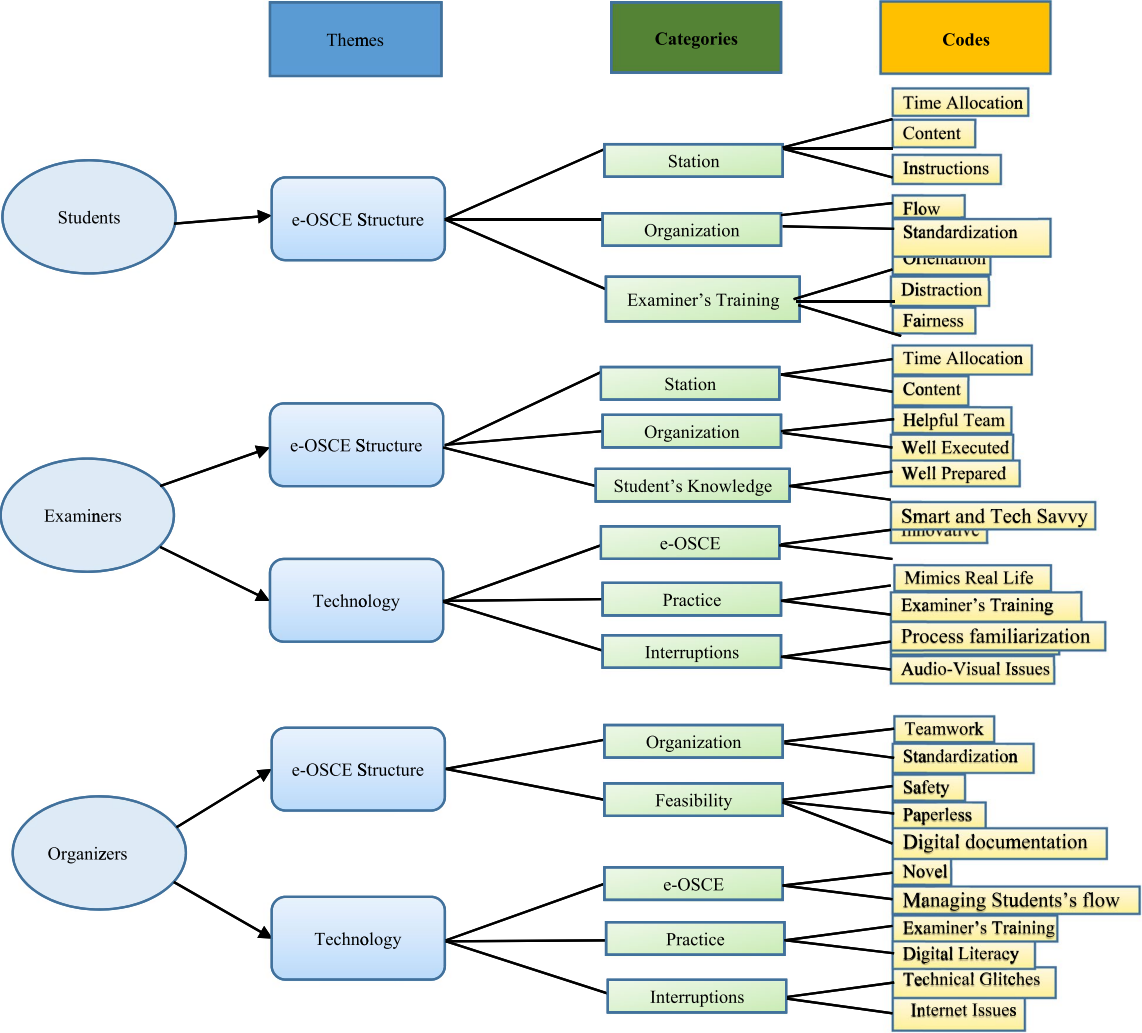
A schematic illustration of the process and outcomes of the qualitative analysis

The process and outcomes of the qualitative analysis are summarized in Fig. 3. As evident in Fig. 3, we gathered responses from students, organizers, and examiners. One theme from students, two themes from examiners and two themes from organizers were generated. In turn, each theme had its own categories and codes. Precisely, two themes of *e-OSCE structure* and *technology* emerged from the data analysis. While e-OSCE structure was a common theme across all study participants, technology usage was not a concern by students. About the *e-OSCE structure theme*, students showed concerns about examiners’ training and adequate pitching of contents.

*“testicular atrophy and infertility are the two well-known complications. As medical students the most common is required from us. if there are other rare complications, it fits urologists to know them better and not medical students”*

” few examiners were extremely slow and it took so much time for them to tell the scenario. Some of them were not familiar with the scenario and that was taking time”.

“I think REST station will be good idea”

Examiners mostly admired the organization, students’ knowledge and digital literacy and showed concerns about time allocation.

"*it is an excellent alternative to the actual OSCE the setup was perfect*"

“I notice really high level for most of the student”

The e-OSCE team observed that the process was well-organized, coherent, and standardized. It was made feasible with a paper-less, synchronous, digital documentation, and safe approach in COVID era.

“*having an alternative platform to conduct OSCE exams was at first something farfetched, but we could achieve it as a team”*

About *technology theme*, interestingly, students did not comment about the advantages or pitfalls of technology. In contrast, the examiners and e-OSCE team commended the innovative and novel aspects of e-OSCE. Digital literacy, internet interruptions and examiners’ training were highlighted by examiners and e-OSCE team.

“*trouble shooting some difficulties the examiners had, a stuck google form, a repeated or not submitted google forms”*

“the calibration session for the examiners should be done before creating google forms”

A comparison of the students’ grades between the last face-to-face OSCE in the pre-COVID era and e-OSCE during the COVID era is presented in Table 3. As evident, students between disciplines. However, students scored lower grades in e-OSCE than in face-to-face OSCE.

**Table 3:** Mean and median scores of students in face-to-face (pre-COVID-19) and e-OSCE (during COVID-19) in medicine II, surgery II and family medicine clinical clerkships

Clinical clerkship	Before COVID-19	During COVID-19	Test^a^	*p*-value
**Family Medicine**				
Number of students	34	35	337	.002
Median Score	88.67	81.94		
Mean score	87.28	82.58		
95% Confidence Interval	85.60–88.96	80.42–84.74		
**Surgery II**				
Number of students	34	34	5.639	< .001
Median Score	90.52	84.58		
Mean (Score)	90.06	84.23		
95% Confidence Interval	88.67–91.44	82.65–85.81		
**Medicine II**				
Number of students	35	35	462.5	0.078
Median Score	90.80	88.11		
Mean Score	88.61	85.53		
95% Confidence Interval	86.60–90.63	82.94–88.13		

^a^Test value is t-test if the data were normally distributed and Mann–Whitney U test if the data were skewed

## Discussion

Our study explicitly demonstrates the utility and efficacy of e-OSCE as a valuable tool in technology-driven assessment. Except for the assessment of psychomotor and procedural skills, e-OSCE enabled us to evaluate the cognitive and metacognitive domains of final year medical students. Furthermore, this work endorses the suitability and effectiveness of e-OSCE for using examiner-based simulation scenarios as an attractive alternative to face-to-face OSCE. In the current tech-savy digital world, our e-OSCE allowed us to assess the desired essential clinical skills of students via distance learning platforms. Lastly, recordings of all stations of e-OSCE provided us a permanent digital repository of the entire exam with the opportunity to review any missed or incomprehensible event. Though a great majority of survey respondents were satisfied with the planning, organization, and implementation of e-OSCE, we observed some challenges including a restricted blueprint, financial expenditures, time-consuming and labour-intensive and the need to train students, examiners, and administrative staff in a short span. The themes of e-OSCE structure and technology were based on the use of digital literacy, training, paper-less exam, coherence, sound organization secure digital repository of exam data. However, there were concerns about stations contents, time allocation, rest between cycles, and relay of instructions.

Using the first level of Kirkpatrick’s model of an event’s evaluation, we have reported the ‘reaction’ of students, examiners and e-OSCE team using online questionnaires [14]. This process underpins the efficacy and suitability of e-OSCE by virtually assessing the final year medical students' clinical skills during the COVID-19 pandemic. Virtual OSCE and e-OSCE are interchangeable terms and, although initial reports have described early success, very few e-OSCEs have been conducted during the COVID-19 era in the MENA region. Worldwide, the investigators have shown a mix of success and challenges from OSCEs that have been organized virtually. In the virtual OSCE, run by Courteille and colleagues, the authors used a virtual patient’s history via texts and videos, video-based examinations and some stations for data analytics [15]. Likewise, Sartori et al., have reported encouraging results of a pilot virtual OSCE on the trainees for telehealth [16]. Similarly, the effectiveness of virtual OSCEs has been documented in the nursing students and in a range of other medical disciplines [17, 18].

Technical feasibility depends on the availability of essential elements required for e-OSCE [19]. In our college, the availability of an online platform, a stable internet, adequate number of computers, headsets, microphones, and cameras allowed us to successfully plan and conduct e-OSCEs. A previous study has shown that the concurrent use of faculty as simulated patients and assessors was acceptable for both students and faculty [20]. In our study, examiners simulated and role-played the given scenarios in all history stations. In contrast to the standard OSCE, examiners rotated among students’ channels, while students permanently stayed in their respective channels. To maintain security and confidentiality of the examination, all virtual movements of students were continuously monitored and recorded via MST. Another layer of security of e-OSCE was provided by synchronous monitoring by examiners in all stations.

In our study, most of the survey responses from students, examiners and organizers recognized smooth organization of e-OSCE. However, we faced challenges in training examiners as well as students in a short time. All stakeholders were unfamiliar with the new format of e-OSCE. Several actions were taken to acquaint them and to minimize technology related issues. Hopwood et al., 2020 have recommended a minimum of three hours of training for students and examiners in order to be acquainted with their roles as well as the logistic of the OSCE [21]. We conducted mock exams that helped us to develop a structured protocol for e-OSCE. The results of the mock exams were slightly different from the real exams and, by and large, there were issues about Internet, equipment familiarity and space for social distancing. Literature has underpinned the value of an initial training session that will help identify any major problems related with technology, equipment or format of the e-OSCE with a follow up training near the assessment time [22]. On the same note, we not only organized early training sessions, to secure standardization in assessment, we also trained examiners of same stations in different panels to reduce interrater variability.

An outright benefit of e-OSCE was its successful completion in less than half of the time than required for standard OSCE. Based on our experience of conducting face-to-face OSCE, the average time needed to examine similar number of students on similar number of stations was approximately five hours. However, each e-OSCE cycle lasted for approximately two and half hours. Moreover, the use of electronic google forms, instead of paper-based sheets, for evaluations saved time spent on adding, auditing, and aggregating marks for each student. Additionally, the submission of electronic evaluation forms by examiners was allowed only when fully completed. Organizers would ensure that each examiner has submitted his grades for each student before the start of the next student’s evaluation. These features eliminated the potential for missing data. In this perspective, Pauline et al., have introduced an online OSCE management information system (OMIS) that integrates stations, items bank, OSCE planning and administration systems and a results analysis software [23]. The OMIS system offers an additional feature of hosting feedback facility which allows the students to receive timely evaluation on their performance. We did not use a customized ready-made platform for e-OSCE. Nevertheless, such platforms offer a range of innovative products that would be valuable for medical educators while assessing clinical competencies of medical students.

The remarks by e-OSCE team “*e-OSCE is time saving, easier for both examiners and students, and save time when dealing with markings*” endorsed our efforts. Although 83.6% of students and 94.1% of examiners acknowledged that e-OSCE ran smoothly, only 21.3% of students and 20% of examiners preferred e-OSCE over face-to-face OSCE. This may be due to the inability of e-OSCE to test physical examination and procedural skills. Some of the responses and comments such as “*thank you for organizing OSCE*”, “*I can see your precious efforts in arranging and preparing the exam as if we are doing it physically in the college*”, “*I believe e-OSCE is an acceptable option in terms of crisis (pandemics) but in normal time I do prefer the traditional face to face*” seem to support the utilization of e-OSCE as an attractive substitute during the pandemic and beyond. In the previous studies, learners who have participated in web-based clinical skill assessment have reported high satisfaction but they have demonstrated preference to face-to-face OSCE as they were concerned for not being able to ‘touch’ patients [24, 25]. Our evaluations also showed similar concerns which reflect an essential element of human behaviours.

In our e-OSCE, we assessed students’ communication, history taking skills and their problem-solving ability without the assessment of physical examination and procedure skills. This important shift in the assessment of clinical skills would necessitate adjusting our future pedagogical strategies to accommodate new skills such as telemedicine consultation which has limited physical examination and focuses mainly on distant disease management [26]. By this, we would be preparing future doctors for a changing health-care delivery where telemedicine is becoming an essential means of delivering a cost-effective, timely and efficient care.

Another added advantage of using technology in e-OSCE, in our study, was the option of reviewing the recorded exam sessions in case of complaint, dispute or incomplete submission of grades by examiners. Video recording during OSCE has been reported in the literature as a means of facilitating the exam and to be a part of the exam itself [27]. Video-taped OSCEs have considerable benefits including quality assurance. However, in the face-to-face OSCE, videorecording would require equipment and expertise to obtain high quality interfaces. In our study, students’ performance was monitored throughout e-OSCE using MST teleconferencing, without the need for additional equipment. After e-OSCE, videos were downloaded and saved for future reference; recorded OSCE stations would give students the opportunity to watch and learn from their performance. This may also be useful for examiner training and for the development of virtual OSCE stations [27].

Incorporating technology in teaching and assessment has many advantages. However, it can pose several barriers. The challenges faced by e-OSCE team were mainly in the preparatory phase for designing, training, resourcing, and securing precautionary measures against the spread of COVID-19. Written responses such as “*having an alternative platform to conduct OSCE exams was at first something farfetched, but we could achieve it as a team*” substantiated the challenge faced by the e-OSCE organizing team. Although, the online platform “MST” was utilized by members of e-OSCE team to conduct synchronous clinical skill sessions, the notion of transferring the set-up of a traditional face-to-face OSCE to a virtual set-up required iterative online meetings and trials with the support of the IT team. Prettyman et al. have emphasized a close collaboration of e-OSCE team with the IT department to ensure the availability and proper function of the computer hardware and software as well as cloud-based systems needed for e-OSCE [27]. Similar degree of collaboration occurred in our study which guaranteed the feasibility and effectiveness of e-OSCE. Previous research with web-based OSCE had shown that students were unsatisfied with the technology and have faced technical difficulties like dropped calls, poor video and audio quality [28]. During our e-OSCE, internet interruptions were reported by students, examiners, and organizers, yet these interruptions were transient and did not disturb the flow of the e-OSCE. All students were assessed as scheduled.

In our study, the students’ performance was lower in e-OSCE than in the traditional face-to-face OSCE in the CoM across all clinical clerkships. Our results are not in conformity with the study by Lara et al., where the researchers did not find difference in mean scores and failure rates between the results of e-OSCE and face-to-face OSCEs [12]. We cannot postulate any scientific reason for low scores in e-OSCE. In order to upskill examiners, structured faculty development programs to supervise, organize and to conduct e-OSCEs using technologies and digital platforms are essential [29, 30]. In our study, most examiners applauded the planning and implementation of e-OSCE and admired the students’ skills in the digital realm. Conversely, students did not comment about the usage of innovative technology-enhanced assessment in e-OSCE. However, they showed minor concerns about the e-OSCE structure and training of examiners. This goes well with the understanding that today’s medical students are digitally native and did not perceive e-OSCE as an advanced intervention. As a quality control, a post-exam item analysis by the assessment committee did not find any issue related to the quality of contents and blueprints. Henceforth, e-OSCE proved feasible with a paper-less, synchronous, digital documentation, and a safe approach in COVID era.

### Study limitations

The e-OSCE model used in our study could not evaluate physical examination and procedural skills of the medical students. This factor limited the scale of assessment of clinical skills.

## Conclusion

This study validates the utility and efficacy of e-OSCE while adhering to the safety precautions for the participants during the COVID-19 pandemic. Overall, the students, examiners and organizers were satisfied with efforts, challenges and accomplishments achieved during the planning, organization, and implementation of e-OSCE. Although e-OSCE was designed to overcome restrictions imposed by the COVID-19 pandemic, this experience has shown substantial advantages applicable to even in the post-COVID era. Though time-consuming and labour-intensive, a sustained program of training and technical support makes e-OSCE suitable, efficient, and cost-effective in assessing clinical competencies. Our e-OSCE reflects an ingenuity in medical education that would open doors for a new paradigm in assessment of future doctors. However, the enigma of assessing physical and procedural skills remains a challenge and further technology-based innovations using artificial intelligence and augmented reality are awaited.

## Supplementary Information

**Additional file 1:** **Appendix 1.** Blueprint of end of clerkship e-OSCE stations. **Appendix 2.** Questionnaire used for the students’ evaluation of e-OSCE. **Appendix 3.** Questionnaire used for the examiners’ evaluation of e-OSCE. **Appendix 4.** Questionnaire used for the e-OSCE team’s evaluation of e-OSCE.

## Data Availability

The datasets used and/or analysed during the current study are available from the corresponding author, Salman Yousuf Guraya, if requested.

## Abbreviations

ACGME: Accreditation Council for Graduate Medical Education
CoM: College of Medicine
COVID-19: Coronavirus Disease 2019
e-OSCE: Electronic Objective Structured Clinical Examination
MENA: Middle East and Northern Africa
OMIS: OSCE Management Information System
