# Supplementary material for: Assessing the utility and efficacy of e-OSCE among undergraduate medical students during the COVID-19 pandemic
Source: BMC Med Educ. 2022 Mar 8;22:156. doi: 10.1186/s12909-022-03218-9 (PMC8902284; doi:10.1186/s12909-022-03218-9)
Supplement: Supplementary file 1 — Additional file 1: Appendix 1. Blueprint of end of clerkship e-OSCE stations. Appendix 2. Questionnaire used for the students’ evaluation of e-OSCE. Appendix 3. Questionnaire used for the examiners’ evaluation of e-OSCE. Appendix 4. Questionnaire used for the e-OSCE team’s evaluation of e-OSCE. [file 12909_2022_3218_MOESM1_ESM.docx]

| **Appendix 1: Blueprint of end of clerkship e-OSCE stations** | | | | | | | |
| --- | --- | --- | --- | --- | --- | --- | --- |
| Panel 1 | Panel 2 | Communication skill | History  taking | Physical examination | Interpretation | Diagnosis | Management |
| **Family Medicine OSCE** | | | | | | | |
| Station 1 | Station 1 | x (explaining result) |  |  | x ( lab result) |  | x |
| Station 2 | Station 2 |  |  |  |  | x | x |
| Station 3 | Station 3 |  | x |  |  | x |  |
| Station 4 | Station 4 |  | x |  |  | x |  |
| Station 5 | Station 5 |  |  |  |  | x | x |
| Station 6 | Station 6 |  |  |  |  | x | x |
| Station 7 | Station 7 | x (breaking bad news) |  |  |  |  |  |
| Station 8 | Station 8 | x |  |  |  |  | x |
| Station 9 | Station 9 |  | x |  |  | x |  |
| **Medicine OSCE** | | | | | | | |
| Station 1 | Station 1 |  | x |  |  | x | x |
| Station 2 | Station 2 |  | x |  |  | x | x |
| Station 3 | Station 3 |  | x |  | x(ECG) | x | x |
| Station 4 | Station 4 | x (counseling & explanation skills ) |  |  |  | x | x |
| Station 5 | Station 5 |  |  |  | x(ECG)(chest x-ray) | x | x |
| Station 6 | Station 6 |  | x |  |  | x | x |
| Station 7 | Station 7 |  |  | x | x (ECG) | x | x |
| Station 8 | Station 8 |  |  | x |  | x | x |
| Station 9 | Station 9 |  |  |  | x (lab result & CT SCAN) | x | x |
| **Surgery OSCE** | | | | | | | |
| Station 1 | Station 1 |  | x |  |  | x | x |
| Station 2 | Station 2 | x (structured oral, explanation) |  |  |  | x | x |
| Station 3 | Station 3 |  | x |  |  | x | x |
| Station 4 | Station 4 |  | x |  |  | x | x |
| Station 5 | Station 5 | x (counseling & explanation skills ) |  |  |  | x | x |
| Station 6 | Station 6 |  | x |  |  | x | x |
| Station 7 | Station 7 |  |  |  | x (video of procedure ) | x | x |
| Station 8 | Station 8 |  |  |  | x (abdominal pelvic x-ray) | x | x |
| Station 9 | Station 9 |  |  |  | x (hand- wrist x-ray) | x | x |

**Appendix 2: Questionnaire used for the students’ evaluation of e-OSCE**

Dear student, the clinical skills team is interested in your feedback about several aspects of the e-OSCE.

Please tick the response that suits you, for each question below. Accurate & objective feedback on the following will be most appreciated:

1. The level of the knowledge and skills tested was overall:

- Too Difficult
- Difficult
- Fair
- Easy
- Too Easy

1. I felt that the virtual OSCE was well organized:

- Strongly Disagree
- Disagree
- Neutral
- Agree
- Strongly Agree

1. I felt that the virtual OSCE ran smoothly:

- Strongly Disagree
- Disagree
- Neutral
- Agree
- Strongly Agree

1. I prefer the virtual OSCE over the traditional face to face OSCE. *

- Strongly Disagree
- Disagree
- Neutral
- Agree
- Strongly Agree

1. Comments and suggestions for improvement.

**Appendix 3: Questionnaire used for the examiners’ evaluation of e-OSCE**

Dear Examiner, the clinical skills team is interested in your feedback about several aspects of the e-OSCE.

Please tick the response that suits you, for each question below. Accurate & objective feedback on the following will be most appreciated:

1. The level of the tested knowledge and skills were over all
   - Too Difficult
   - Difficult
   - Fair
   - Easy
   - Too Easy
2. I felt that the virtual OSCE was well-organized.
   - Strongly Disagree
   - Disagree
   - Neutral
   - Agree
   - Strongly Agree
3. I felt that the virtual OSCE ran smoothly.
   - Strongly Disagree
   - Disagree
   - Neutral
   - Agree
   - Strongly Agree
4. I prefer the virtual OSCE over the traditional face to face OSCE.

- Strongly Disagree
- Disagree
- Neutral
- Agree
- Strongly Agree

1. Comments and suggestions for improvement.

**Appendix 4: Questionnaire used for the e-OSCE team’s evaluation of e-OSCE**

Dear OSCE team

Thank you for sharing your experience with the e-OSCE by completing the form.

1. Describe the most beneficial aspect of your experience with the e-OSCE?
2. What are the most challenging issues you have experienced while preparing for the e-OSCE?
3. What are the most challenging issues you have experienced DURING the e-OSCE?
4. What are the most challenging issues you have experienced AFTER the e-OSCE?

Kindly provide your suggestions for improvement of future e-OSCEs?
